# Use of ergogenic aids among Brazilian athletes: a cross-sectional study exploring competitive level, sex and sports

**DOI:** 10.3389/fspor.2023.1257007

**Published:** 2023-09-22

**Authors:** Géssyca T. de Oliveira, Hiago L. R. de Souza, Anderson Meireles, Marcelo P. dos Santos, Laura H. R. Leite, Renato M. Ferreira, Moacir Marocolo

**Affiliations:** ^1^ Human Physiology and Performance Research Group, Department of Physiology, Institute of Biological Sciences, Federal University of Juiz de Fora, Juiz de Fora, Brazil; ^2^ Aquatic Activities Laboratory, Department of Physical Education, Federal University of Ouro Preto, Ouro Preto, Brazil

**Keywords:** athletic performance, ergogenic substance, exercise, sports, athletes

## Abstract

Was investigated ergogenic aids (EAs) used by Brazilian athletes and their association with performance, sex, sports classification, and modality. It identified the main purposes of EAs and their prescription.

**Methods:**

239 athletes of 15 modalities, ranging from regional to international level, answered a survey online.

**Results:**

Highly competitive athletes consumed nutritional and mechanical aids more (OR = 1.96 CI 95% [1.28–2.9]; OR = 1.79 CI 95% [1.29–2.47]), while the use of psychological EAs decreased [OR = 1.66 95% CI (1.18–2.94); *p* = 0.001]. Male athletes [OR = 1.44 CI 95% (1.11–2.88)] and individual sports practitioners [OR = 1.78 CI 95% (1.02–3.11)] used nutritional aids more. Triathlon athletes had higher nutritional EA use, while soccer athletes had lower. Combat sports athletes had higher pharmacological EA use.

**Conclusion:**

Athletes use nutritional and pharmacological aids more to improve performance and gain lean body mass. Mechanical aids were used for recovery and psychological aids for motivation. Self-prescription is common, especially for pharmacological aids.

## 1. Introduction

Maximizing performance is a complex and multi-faceted endeavor that involves more than just training. Other important factors such as nutrition and mental ability play a crucial role in determining an athlete's overall success (1). In this context, the judicious use of ergogenic aids (EAs) can make a significant impact on an athlete's training and competition outcomes. These resources are specifically designed to improve exercise performance, optimize training adaptations, and support post-exercise recovery, and are an integral part of the training routine for many athletes (1).

EAs are classified into (1) mechanicals: tools, clothing, and devices that can induce a physiological response and contribute to the performance of physical capacities (i.e., ischemic preconditioning, compression stockings, and swimsuits (2); (2) nutritional: dietary supplements made of ingredients that are effective in promoting further increases in energy substrate stores, and provoking muscle hypertrophy or training performance enhancement (i.e., creatine supplements and caffeine) (3); (3) psychological: strategies that have the potential to modulate behavior, optimizing concentration and motivation (i.e., hypnosis and music) (4); and (4) pharmacological: drugs that induce a supraphysiological increase in substances or hormones that can contribute to the repair of body tissues and muscle growth and, consequently, an improvement on physical performance (i.e., anabolic steroids) (5).

In the sports context, the use of EAs may be legal or illegal, involving both benefits and risks. Its improper use can cause adverse effects on the athlete's health, in addition to unintentional doping caused, for example, by the ingestion of nutritional aids containing substances prohibited by the World Anti-Doping Agency. The concern about the use of these resources also relies on the lack of knowledge about their factual contributions to performance. There is conflicting evidence about the effects of EAs on exercise performance, which may vary between the characteristics of sports modalities, predominantly on energetic pathways (aerobic or anaerobic), between sexes (6, 7) and fitness level of athlete, since EAs responses may be trivial among amateur athletes, but significant among high-performance athletes.

The growing search for adjuvants capable of enhancing physical performance, coupled with the lack of studies that address the four categories of EAs, was the premise for the present study to be developed. Most studies focus primarily on nutritional and pharmacological aids, limiting our understanding of other types of aids. Furthermore, investigations often restrict their scope to individuals in gym settings and individual sports, making it difficult to apply the findings to diverse sports such as triathlon and modern pentathlon. By investigating the prevalence of EAs use among athletes, this study aims to gain insights into the motivation for use and identify trends. This knowledge will inform targeted education and policies to support informed decision-making to promote the safe and effective use of EA and optimize athlete performance and well-being.

Thus, the objectives of the study are to investigate the prevalence of the use of EA among athletes in Brazil, explore associations with the competitive level, sports classification, modalities, and sex, understand the motivations of athletes for the adoption of aids and identify means of prescription.

## 2. Materials and methods

This is a cross-sectional, quantitative, and exploratory study conducted between August 2021 to April 2022. The study was approved by the Local Ethics Committee (n° 4.120.625) and carried out in full accordance with the declaration of Helsinki.

### 2.1. Subjects

Two hundred and thirty-nine Brazilian (134 male and 105 female) competitive athletes from regional to international levels and several sports modalities volunteered in the study. Athletes from fifteen sports were identified swimming (*n* = 44), track and field (*n* = 32), soccer (*n* = 32), triathlon (*n* = 25), handball (*n* = 19), cycling (*n* = 17), combat sports (*n* = 13), basketball (*n* = 12), trampoline gymnastics (*n* = 11) volleyball (*n* = 9), modern pentathlon (*n* = 9), rugby (*n* = 4), shooting sports (*n* = 4), weightlifting (*n* = 4), and American football (*n* = 4). The inclusion criteria were: (a) minimum age of 13 years old (underage consent was required from a legal tutor); (b) having participated in competitions and systematized training in the last 3 years; (c) providing consent to participate in the study; and (d) having completed the proposed questionnaire. Demographic and anthropometric characteristics of volunteers are presented in Table 1.

**Table 1:** Anthropometric characteristics and competitive level of athletes.

	Female	Male	Total
Athletes	105	134	239
Age (years)^a^	24.6 ± 8.2	28.4 ± 9.8	26.7 ± 9.3
Body mass (kg)^a^	62.6 ± 11.8	78.7 ± 36.3	70.02 ± 14.0
Height (cm)^a^	164.3 ± 7.5	175.1 ± 7.8	170.4 ± 9.4
Competitive level	43.9%	56.1%	100%
Regional	47 (44.8%)	46 (34.3%)	93 (38.9%)
State	28 (26.7%)	32 (23.9%)	60 (25.1%)
National	23 (21.9%)	43 (32.1%)	66 (27.6%)
International	7 (6.7%)	13 (9.7%)	20 (8.4%)
Type of sport	*N* (%)	*N* (%)	*N* (%)
Individual	73 (69.5%)	91 (67.9%)	164 (68.6%)
Collective	32 (30.5%)	43 (32.1%)	75 (31.4%)

^a^ Data are presented as mean ± SD.

### 2.2. Data collection

The survey link was shared by e-mail and social media such as Instagram®, WhatsApp®, and Facebook®. In the questionnaire, the athletes were informed that their participation would be voluntary and anonymous and that they could suspend it at any time. To minimize the reliability bias of the answers, an average duration of 10 min was estimated for the athletes to answer the questions (8).

### 2.3. Instruments

The survey was developed by a team of experienced researchers and sports performance experts, considering references (9, 10). It was conducted using an online research platform (Google Forms®) and underwent pilot tests to identify potential errors.

The survey consisted of 55 questions in Portuguese language and covered participants' demographic data, current or past use of ergogenic aids (EAs), and prescription methods. Most questions were presented in multiple-choice format, some accompanied by a Likert scale. A few questions required a written response. Athletes were not obligated to answer all the questions, and the “other” response option was available for most multiple-choice questions. The questionnaire was designed to skip irrelevant questions based on previous responses and redirect the respondent to a specific section.

The questions were distributed into 14 sections: (a) ethical aspects and study objectives, along with anthropometric information and competition level; (b) training routine-related questions, such as number of sessions per week, duration, practice time, and individual goals; (c) prescription methods, frequency, and reasons for EA use. Questions about each type of EA were grouped into specific sections, preceded by a single question, such as “Do you use nutritional ergogenic supplements?”. Athletes were directed to the corresponding section based on their responses. The sections included definitions and examples of EAs to facilitate understanding. The snowball sampling technique was employed, allowing volunteers to indicate another athlete to participate in the research at the end of the questionnaire (11).

### 2.4. Statistical analysis

The data were classified according to observed frequencies and grouped by competitive level, sex, sports classification, and modality. Was determine frequencies for qualitative variables and means and standard deviations for quantitative variables. Pearson's chi-square test was used at a 5% significance level. *Post hoc* Goodness-of-fit test was used for contingency tables with an effect size of 0.5, an alpha (α) error of type I error probability of 0.05, a total sample size of 239, and a degree of freedom (df) equal to 1. The non-centrality parameter λ, indicating the strength of the effect, was 59.75. A critical value of *χ*² at 3.84 was used to reject the null hypothesis. The power achieved was 1.00. Phi coefficient and Crame's *V* were used to evaluate the strength of the relationship between the variables, with values of >0 = No or very weak, >0.05 = Weak, >0.10 = Moderate, >0.15 = Strong, and >0.25 = Very strong (12). The Odds Ratio was calculated as a measure of association between categorical variables, obtained through a logistic regression model. The Shapiro–Wilk test was used to verify the normality of the sample characterization variables' distribution. The IBM SPSS Statistics 64-bit version 25.0 was used for association analyses.

## 3. Results

### 3.1. Use of EAs and competitive level, sex, and sports classification

Of the total athletes, 62.8% use nutritional, 29.7% use mechanics, 7.1% and 44.4% use pharmacological and psychological aids. At the low competitive level, nutritional aids account for 54.9% of the total, while mechanical, pharmacological, and psychological aids correspond to 22.2%, 5.9%, and 37.7%, respectively. In the highly competitive level, nutritional is the most utilized, representing 76.7%, followed by mechanical (43.0%), pharmacological (9.3%), and psychological aids (57.0%).

Men athletes' have a higher proportion of aid usage in all categories, with nutritional aids being the most utilized (70.9%), followed by mechanical (32.8%), pharmacological (8.2%), and psychological aids (45.5%). Female athletes show a higher preference for nutritional aids, with a utilization rate of 52.4%, followed by mechanical (25.7%), pharmacological (5.7%), and psychological aids (42.9%) (Table 2).

**Table 2:** Relation of the use of EAs by competitive level and sex.

Ergogenic aids				
Competitive level	Nutritional (%)	Mechanical (%)	Pharmacological (%)	Psychological (%)
Low	54.9	22.2	5.9	37.7
High	76.7	43.0	9.3	57.0
Female	52.4	25.7	5.7	42.9
Male	70.9	32.8	8.2	45.5

The use of EAs and the competitive level showed a significant association. For the use of nutritional EAs [*χ*² = 11.238; gl = 1; phi = 0.22, OR = 1.96 95% CI (1.28–2.9); *p* < 0.001], most of the affirmative answers (56%) occurred in the highly competitive level group, while most negative responses (77.5%) occurred in the low competitive level group. High-performance athletes are more likely (OR = 1.96) to consume nutritional aids. Likewise, with the use of mechanical EAs [*χ*² = 11,407; gl = 1; phi = 0.22, OR = 1.79 95% CI (1.29–2.47); *p* = 0.001], most responses affirmative answers (52.1%) occurred in the highly competitive level group, while most of the negative answers (70.8%) occurred in the low competitive level group. High-performance athletes are more likely (OR = 1.79) to use mechanical aids. For the use of psychological EAs [*χ*² = 8.676; gl = 1; phi = −0.19, OR = 1.66 95% CI (1.18–2.94); *p* = 0.001], although 57% of high-performance athletes use psychological AEs and 46% of low competitive level athletes use these resources, the negative phi coefficient indicates an inverse association between the variables. Therefore, together with the OR, it seems that as the competitive level increases, the probability of individuals reporting the use of psychological EAs decreases by 1.66. The association between low and highly competitive levels and the use of pharmacological EAs was not significant (*χ*² = 0.975; gl = 1; phi = −0.64, *p* = 0.32).

The use of EAs about the sex of the athletes showed an association only for the use of nutritional EAs [*χ*² = 8.635; gl = 1; phi = −0.19, OR = 1.44 95% CI (1.11–2.88); *p* = 0.003], with a higher percentage of male athletes (63.3%) reporting the use compared to female athletes (36.7%). The OR showed an of 0.452, indicating that female athletes are less likely to use nutritional EAs, unlike male athletes, who were a 1.445 times higher probability to use this feature.

In the analysis by sports classification, a significant association was observed only for the use of nutritional EAs [*χ*² = 4.157; gl = 1; phi = 0.31, OR = 1.78 95% CI (1.02–3.11); *p* = 0.04]. Athletes who practice individual sports are a 1.21 times higher probability to use nutritional EAs when compared to athletes in team sports. Regarding the modality, in the analysis of the subgroup, the triathlon athletes presented frequencies observed above the expected for the use of nutritional EAs (Cramer's *V* = 0.31; *p* = 0.00), and the soccer athletes presented observed frequency below the expected (Cramer's *V* = 0.31; *p* = 0.00). Athletes practicing combat sports observed frequencies higher than those expected for the use of pharmacological EAs (Cramer's *V* = 0.33; *p* = 0.00).

### 3.2. Reasons for the use and means of prescription of EAs

**GRAPH 1:**
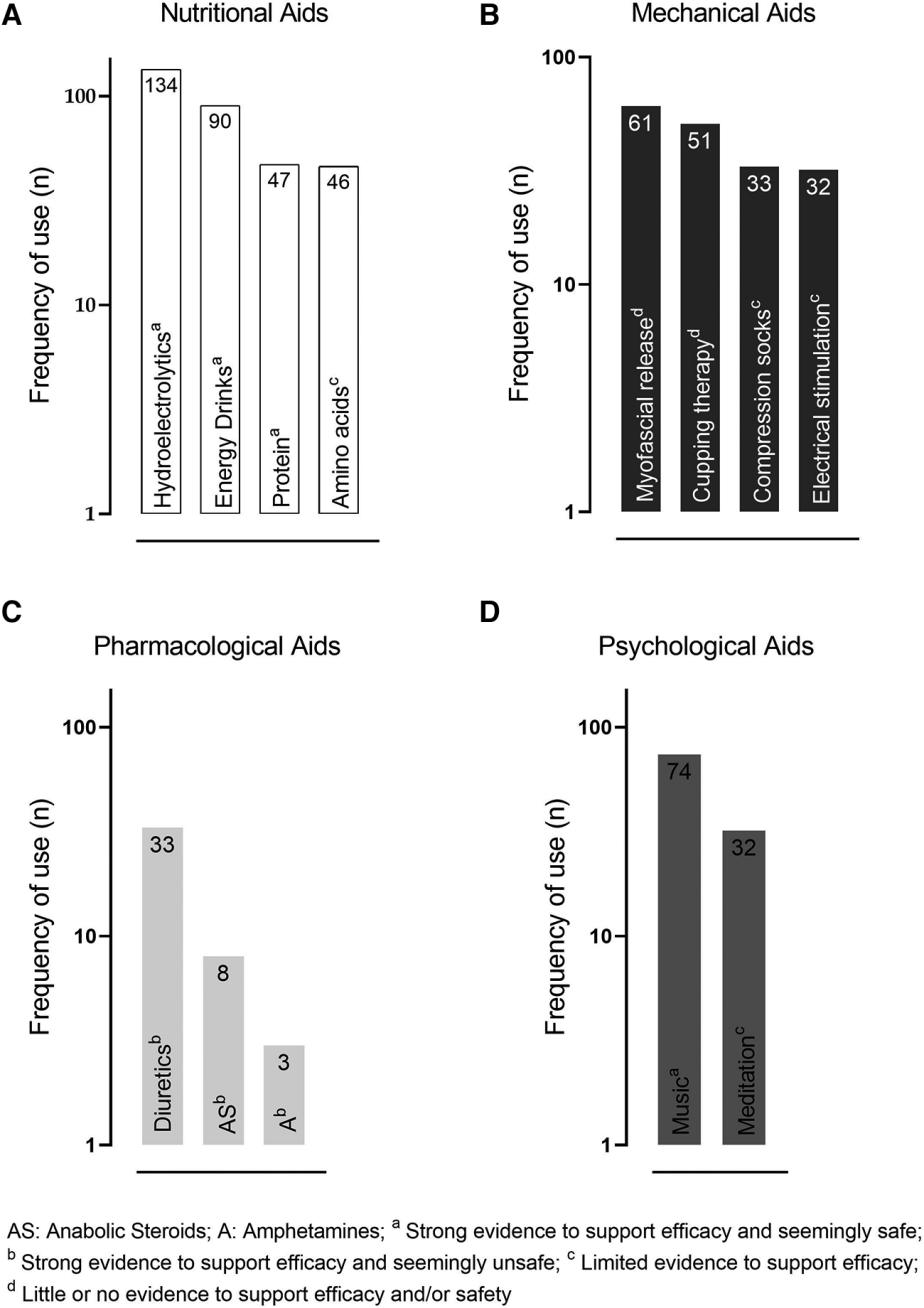
Frequency of citation of use of each EA. Types of ergogenic aids most used by athletes.

Nutritional and pharmacological aids were predominantly chosen by athletes focusing on improving exercise performance (76% and 58.8% respectively) and gaining lean mass (24% and 41.2%, respectively). For mechanics aids, recovery was the reason for 70.4% of participants, followed by 15.5% for performance improvement and 14.1% for injury prevention. Mechanical aids were the preferable option choice of athletes focusing on improving recovery (70.4%) and exercise performance (15.5%). Finally, enhancing motivation and concentration were the reasons for using psychological aids (50.9% and 49.1%, respectively) (Graph 1).

Professional prescription of nutritional aids was prevalent among nutritionists (52.7%), followed by doctors (16.4%), trainers (5.5%), and physical therapists (5.5%). Mechanical aids prescription was mostly provided by physical therapists (74.1%) doctors (7.4%) and trainers (3.7%). Psychological aids were prescribed by psychologists (24.4%) and trainers (8.9%) predominantly. Fifty percent of pharmacological aids prescriptions were performed by doctors.

Self-prescription counted for 20%, 14.8%, 62.2%, and 50% of nutritional, mechanical, psychological, and pharmacological aids usage, respectively. A friend's recommendation influenced the use of nutritional (5.5%) and psychological aids (4.4%) by athletes.

## 4. Discussion

This study aimed to investigate the prevalence of EAs use by athletes in Brazil and identify an association between competitive level, sports classification, sports modality, sex, and reasons for use and means of prescription. Results showed that higher competitive level athletes are more likely to use EAs, with nutritional aids as their preferred choice. Male athletes in individual sports, especially triathlon, are more likely to consume nutritional aids, while team sports athletes like soccer players have less preference. Combat sports athletes prefer pharmacological aids. The reasons for using EAs were to improve exercise performance, recovery, and motivation. Psychological and pharmacological aids were mostly self-prescribed. These findings suggest that EAs use is influenced by sex, competitive level, sports classification, and sports modality, mainly for performance enhancement and recovery.

The current data shows that the use of EAs increases according to the competitive level of the athletes. Specifically, a prevalence of nutritional aid use was observed. These results corroborate the meta-analysis by Lun, Erdman (13) who observed that 81%–100% of a sample of 440 high-performance Canadian athletes adopt nutritional supplementation, of whom 76% were competing internationally. As previously demonstrated (3, 14) male athletes use more nutritional aids compared to female athletes. However, it has already been observed, and no difference between the sexes (7). Such contradictory results may be explained by methodological differences such as sample size and the sports modality investigated. Although in this study there was no association between the use of other types of EAs and the sex of athletes, the percentage of their use was prevalent among male athletes as well.

Athletes of higher competitive levels (national and international) also greatly adopt mechanical aids. At the highest levels of competition, all athletes are highly trained and the difference between success and defeat is marginal. Therefore, the use of strategies to improve training outcomes can be decisive. In fact, in a real-world setting, athletes are likely to use more than one EA at repeated times and days. It is proposed that EAs usage by highly competitive athletes represents their last strategy to maintain themselves at a highly competitive level (15, 16).

It is interesting to note that there is an inverse association between the use of psychological aids and competitive level. Athletes at higher competitive levels may have more access to specialized resources, such as sports psychologists, who can provide personalized techniques, making psychological aids less necessary. Furthermore, higher-level athletes may have greater experience to deal with the psychological demands of sport, making psychological aids less useful (17). Studies have shown that both music and meditation can have positive effects on reducing stress, anxiety, and improving focus and performance in sports (18, 19).

The use of pharmacological aids did not show a significant association with competitive level, indicating other factors influencing their use. Additionally, it is important to acknowledge that the use of performance-enhancing substances is prohibited in most sports and can have serious health consequences, so the findings of this study should not be taken as an endorsement of their use.

Each sport has unique characteristics and different metabolic needs; therefore, different EA may vary between them. Most of the research are concentrated on the use of nutritional aids, mainly individual sports athletes (20–23). Triathlon athletes, for example, use more nutritional aids probably due to being a sport that encompass three modalities that demand high energy expenditure both in training and in competitions (24). In contrast, combat sports athletes, consume pharmacological aids, with preference to the consumption of diuretics (25). It is well known that these athletes need to quickly adapt their body mass to the competitiońs weight category, which are usually below their routine weight (26). On other hand, team sports such as soccer may use less EA. One possible reason may be that in team sports, athletes' performance may depend more on teamwork, strategy, and tactics than on individual factors such as strength or endurance. Therefore, the use of EA may have less impact on the performance of athletes in team sports than in individual sports (27).

Several reasons can be punctuated for the use of the EAs. For nutritional aids, performance optimization was reported higher, as was for using pharmacological aids. Recovery and motivation were the main reasons for using mechanics and psychological aids, respectively. Which shows that athletes are always looking for a strategy that can provide a competitive advantage. That said, due to the characteristics of our sample, it is important to consider that beginner athletes may have greater restrictions on accessing information about the use of strategies to optimize performance, due to financial restrictions of the sport, geographic issues, among other reasons, on the contrary athletes of a higher competitive level (28).

Herein, it was demonstrated that the prescription of EAs is not always conducted by an accredited professional. Nutritional aids, for example, which had the highest percentage of use among athletes, were also prescribed by doctors and trainers. For all types of EAs, self-prescription reports were surprisingly high, reaching 20% for nutritional and 50% pharmacological aids (13, 29). Athletes are often exposed to pain, fatigue, injuries, and recovery difficulties, which could persuade the search for immediate solutions. In this sense, according to Baltazar-Martins, Brito de Souza (28), athletes seem to be unaware of adequate sources of information, contributing to the involuntary ingestion of substances that may have acute and long-term side effects, in addition to doping.

This study has some limitations. Since date of data collection was during the COVID-19 pandemic this report of EAs use should take into consideration adapted training/competition routines to the lockdown scenario. Nevertheless, any deviation in the EAs outcomes is inherent to studies with similar design. Open-ended and multiple-choice questions were used to reduce recall inaccuracies. Furthermore, although anonymous and voluntary participation was emphasized, some athletes may have avoided reporting some information about EAs use. Finally, it was restricted to analyze the global prevalence and patterns of use of EAs among athletes.

Some studies (25, 30) have already pointed out that the uncontrolled use of EAs can contribute to the search for illicit resources. The use of lawful and illicit EAs permeates the sport at all competitive levels and the risk factors for athletes to start adhering to substances characterized as doping are indeterminate, which hinders prevention efforts. Tied to this, research on the real contribution of EAs most used by athletes seems to be necessary. Demystifying athletes' beliefs and expectations in the face of the ergogenic influence of a resource on physical performance can contribute to well-informed decisions being made about the use of auxiliaries to improve exercise performance.

## Data Availability

The raw data supporting the conclusions of this article will be made available by the authors, without undue reservation.
